# T Cell-Tumor Interaction Directs the Development of Immunotherapies in Head and Neck Cancer

**DOI:** 10.1155/2010/236378

**Published:** 2010-12-27

**Authors:** A. E. Albers, L. Strauss, T. Liao, T. K. Hoffmann, A. M. Kaufmann

**Affiliations:** ^1^Department of Otolaryngology, Head and Neck Surgery, Charité-Universitätsmedizin Berlin, Campus Benjamin Franklin, 12200 Berlin, Germany; ^2^Fondazione Humanitas per la Ricerca, 20089 Rozzano, Italy; ^3^Department of Otolaryngology, Head and Neck Surgery, Universität Essen, 45147 Essen, Germany; ^4^Department of Gynecology, Charité-Universitätsmedizin Berlin, Campus Benjamin Franklin and Campus Mitte, 12200 Berlin, Germany

## Abstract

The competent immune system controls disease effectively due to induction, function, and regulation of effector lymphocytes. Immunosurveillance is exerted mostly by cytotoxic T-lymphocytes (CTLs) while specific immune suppression is associated with tumor malignancy and progression. In squamous cell carcinoma of the head and neck, the presence, activity, but also suppression of tumor-specific CTL have been demonstrated. Functional CTL may exert a selection pressure on the tumor cells that consecutively escape by a combination of molecular and cellular evasion mechanisms. Certain of these mechanisms target antitumor effector cells directly or indirectly by affecting cells that regulate CTL function. This results in the dysfunction or apoptosis of lymphocytes and dysregulated lymphocyte homeostasis. Another important tumor-escape mechanism is to avoid recognition by dysregulation of antigen processing and presentation. Thus, both induction of functional CTL and susceptibility of the tumor and its microenvironment to become T cell targets should be considered in CTL-based immunotherapy.

## 1. Introduction

Squamous cell carcinomas of the head and neck (SCCHN) are the sixth most frequent type of malignancy worldwide. SCCHN accounts for approximately 6% of all cancer cases and for about 650,000 new cases and 350,000 deaths worldwide each year [1–3]. While early-stage SCCHN can be treated relatively effectively, fewer than 40% of patients with advanced, metastatic disease can be cured. Unfortunately, about two thirds of patients with SCCHN present with advanced-stage disease, commonly involving regional lymph nodes. Distant metastases are found in about 10% of patients at initial presentation. The 5-year survival for all stages is about 60%. Despite significant improvements in surgery, radiation, and chemotherapy, long-term survival rates in patients with advanced stage SCCHN have not significantly increased in the past 30 years [4–6]. 

Mortality from SCCHN remains high because of the development of distant metastases and the emergence of therapy-resistant local and regional recurrences. It is therefore essential to develop a deeper understanding of the biology of this disease for more effective alternative therapies such as immunotherapy. As basis for immunotherapeutic approaches, interactions between tumors and the host immune system have been a subject of many studies. It has been shown that a naturally induced T-cell response recognizing SCCHN exists that could potentially target and possibly kill the tumor cells. Most cases in which this may have happened will remain obscure because they never become visible. Those cases that become clinically apparent show a different constellation. On one hand, antitumor immune effects can be observed; on the other, a deleterious effect on immune cells is exerted by the tumor. Tumor progression itself is therefore invariably linked to selective and pervasive impairment of immune cells. 

The identification and characterization of a variety of human tumor antigens with possible use for immunotherapy and immunomonitoring [7] and expectations triggered by successful in vitro tests and therapeutic results in animal models have led to rapid translation of these experimental findings into clinical testing. This resulted in a rather large number of patients with various types of malignant diseases having been enrolled in clinical trials of T cell-based immunotherapy. In many cases tumor antigen-specific CTL immune responses could be achieved and successfully monitored, but unfortunately these findings did not correlate with clinical responses [8]. True clinical responses attributable to immunotherapy have been sparse so far. This discrepancy has initiated investigations into mechanisms underlying the failure of tumor antigen-specific CTL to control tumor growth in cancer patients, especially those treated with immunotherapies. The mechanisms responsible for this impairment may vary depending on the nature of the tumor milieu and manifest in the tumor microenvironment as well as in the periphery [9, 10].

For the development of strategies to prevent or reverse tumor-induced effects and to protect immune cells in the tumor microenvironment, studies of both (a) naturally occurring immune cells that could be recruited for immunotherapeutic strategies targeting specifically the tumor cells and (b) direct and indirect mechanisms responsible for dysfunction and death of immune cells in SCCHN are essential [11, 12]. 

This review focuses on the mechanisms of tumor-mediated interference with the host immune system and CTL in particular concerning SCCHN. We describe tumor-escape mechanisms from the immune system at the tumor site and in the periphery and describe strategies to redirect the immune system to a more effective antitumor response.

## 2. Distinct Etiologies of Head and Neck Cancer

Two distinct causes of SCCHN are known. SCCHN is either caused by the spontaneous accumulation of multiple genetic alterations modulated by genetic predisposition and chronic inflammation, enhanced by environmental influences such as tobacco and alcohol abuse or by infection with oncogenic human papillomavirus (HPV). Carcinogens are regarded the most important factors. Thus, two main etiologies can be defined: tumors induced by toxic substances or by the activity of the viral oncogenes of HPV. Both etiologies involve a multistep process and result in alterations affecting two large groups of genes: oncogenes and tumor suppressor genes. However, it has been demonstrated that another set of genes is also altered. These are related to immune response mechanisms like the MHC complex. This observation is important because it shows that immune recognition is relevant and probably a force driving selection in the tumor towards immune-resistant variants (see below).

HPV-associated SCCHN defines a distinct subgroup that could specifically be targeted by novel preventive or therapeutic measures. More than 100 human papillomavirus (HPV) subtypes are known to date. Of these, 15 have been shown to be oncogenic in humans. HPV type 16 and 18 seem to play the major role in the etiology of HPV an associated SCCHN, particularly those arising in the oropharynx [13, 14]. HPV infection has been detected in 20% to 30% of tumors located in all head and neck anatomic subsites and in about 50% of tonsil squamous cancers. For laryngeal cancer, the role of HPV is less clear. Data on the prevalence of high-risk HPV-associated SCCHN vary and multicenter studies have not been performed yet [15, 16]. 

The lower rate of carcinogenic-risk factors and p53 mutations and a younger patient population suggests that factors, currently unknown, are associated with viral entry, propagation/transformation, and immune evasion in HPV-associated SCCHN patients [14, 17]. Failure to clear HPV infection leaves host cells under the influence of the viral oncogenes. Persistently infected persons can develop clinically or histologically recognizable precancers that can persist and may develop over time into invasive cancer. These oncogenes are vital to the tumor cell survival and proliferation and therefore provide a suitable target for anti-tumor vaccination. 

Therefore, with these two different etiologies, different treatment options according to the genesis of their malignancy may be developed for future patients. Immunogenic tumors in patients mounting specific immune responses may be treated by induction or enhancement of specific CTL responses. Such responses may have clinical impact for primary therapy or in adjuvant settings when tumor burden has been reduced.

## 3. Immunoresponses in SCCHN

### 3.1. Tumor-Specific T Cells

Effective antitumor responses in individuals with SCCHN depend on the presence and function of immune cells that are able to recognize and eliminate tumor cells. These tumor antigen-specific T cells are known to be present in the peripheral circulation and tissues of patients with cancer. They can be monitored by multicolor flow-cytometry directly using tetrameric peptide-MHC class I and II complexes, so called tetramers. These molecules bind to the cognate T cell receptor. These stainings are combined with T cell markers (e.g., CD3, 4, 8) and if desired with makers for the differentiation (e.g., CCR7, CD45RA) and the functional (e.g., CD107a, perforin, granzyme B) or dysfunctional (e.g., annexin, 7-AAD, CD3-zeta-chain) status of these cells. 

A number of studies have investigated the frequency of tumor-specific T cells in the peripheral circulation of patients and in the tumor [9, 10, 18–24]. In most of these studies, T cells reactive against p53-derived epitopes by HLA-class-I and II were described. The special interest in p53-derived epitopes can be explained by the fact that the majority of human cancers, including head and neck cancer, seem to “overexpress” this protein. Other studies have focused on T cells specific to HPV-derived epitopes [25]. 

Wild-type (wt) sequence p53 peptides like other tumor epitopes are processed and presented to the host immune cells either directly by the tumor cells or by professional antigen presenting cells (APCs) such as dendritic cells (DC). This results in an increased number of wt p53 peptide-specific T cells and, in some instances, p53-specific antibodies [21, 26, 27]. 

It could be demonstrated that the frequency of tetramer^+^ CD8^+^ T cells specific for the HLA-A2.1-restricted wt p53_264-272_ peptide is significantly higher in the peripheral circulation of HLA-A2.1^+^ patients with head and neck cancer than that in normal donors [27]. In a subsequent study, the frequency of wt p53 epitope-specific T cells for two distinct epitopes was determined in the peripheral circulation and in tumor infiltrating lymphocytes (TIL) isolated from SCCHN. Wt p53 epitope-specific T cells were found to be significantly enriched in the TIL, demonstrating a preferential localization at the tumor site or in tumor-involved lymph nodes [9]. Interestingly, the presence and frequency of wt p53 epitope-specific effector T cells among TIL did not correlate with tumor stage. This implies that the frequency of tetramer^+^CD8^+^ effector cells alone has no effect on tumor progression. In this study, two patients with sufficient numbers of TIL were available to test in vitro responsiveness after polyclonal stimulation with anti-CD3 mAb. Only a low IFN-*γ* expression of the CD3^+^CD8^+^ T cells could be measured indicating a poor responsiveness to this stimulus. At the same time, a significantly increased number of regulatory T cells (Treg) were found at the tumor site compared to the periphery [9]. It has been well accepted that the presence and accumulation of Treg inhibits T-cell responses in vivo and may be responsible in part, for downregulation of antitumor immune responses in patients with head and neck cancer [28]. Treg are likely to mediate suppressive effects directed at self-reactive T cells (see below in detail) [29]. This immunosuppressive mechanism may be particularly relevant to T cells that recognize self-peptides like wt p53 epitopes and thus are likely to be tolerized, especially at the sites of their accumulation in tumor tissues. Other data also confirm the depressed functionality or even spontaneous apoptosis of CD8^+^ tumor-specific T lymphocytes [10]. To date, the molecular mechanisms driving spontaneous apoptosis of circulating epitope-specific T cells in patients with cancer are unknown. Different and from each other independent factors and mechanisms like the Fas/FasL pathway, propriocidal lymphocyte death [30], or tumor-derived factors responsible for disruption of signal transduction pathways may play a role. The recent discovery of FasL^+^ microvesicles in the circulation of patients with head and neck and other cancers also suggests a potential mechanism for systemic elimination of CD95^+^ activated CD8^+^ T cells. However, neither the tumor origin of microvesicles present in patients' sera nor their in vivo participation in T cell apoptosis has been confirmed. 

A subgroup of head and neck cancer is associated with human papilloma virus. Virus-derived antigens are considered superior targets for T cells than tumor-associated self-antigens because they have higher affinity to MHC and are more immunogenic. As opposed to tumor-associated self-antigens, thymic negative selection of T cells recognizing these virus derived antigens has not reduced the pool from which tumor reactive T cells can be recruited. Therefore, HPV-encoded oncogenic proteins, such as E6 and E7, are promising tumor-specific antigens in addition to the fact that they are considered obligatory for tumor growth. Surprisingly, few studies have characterized endogenous T cell immunity to HPV-encoded oncogenes in SCCHN as a prerequisite for immunotherapeutic targeting of these antigens. Two studies show an increased frequency of CD8^+^ T lymphocytes directed against HPV E7 epitopes documenting a natural immune response [18, 22]. These HPV-specific T cells were able to recognize and kill a naturally HPV-16 transformed SCCHN cell line after IFN-*γ* treatment that enhanced antigen processing and presentation by the tumor cells. Further phenotypic characterization of the HPV-specific T cells revealed an increase in terminally differentiated/lytic T cells (CD8^+^CD45RA^+^CCR7^−^). This population was also characterized by a high frequency of staining for the degranulation marker CD107a in E7 tetramer^+^ T cells, compared with bulk CD8^+^ T cells, consistent with their terminally differentiated lytic, degranulated status. These cells may account for the unsuccessful antiviral immune response [31] to these tumors, indicating that incomplete activation of tumors-pecific T cells or suboptimal target recognition may enable tumor progression in vivo. On the other hand, if T cells from this reservoir could be adequately activated and expanded, these cells could provide a valuable reservoir of effectors for cancer vaccination. 

Several current studies suggest that cancer vaccines have increased efficacy if they incorporate tumor-specific cytotoxic as well as helper epitopes. Although CTL are considered to play the primary role in tumor eradication, it is also hypothesized that the participation of tumor antigen-specific CD4^+^ T-helper lymphocytes may be required for optimal antitumor effects by generating and maintaining antitumor immune responses through interactions with CTL and other cells [32, 33]. As a result, efforts have been made to define class II HLA-restricted tumor peptides for use in cancer vaccines. As for CTL defined epitopes, wild-type sequence (wt) p53 peptides also provide a source for CD4^+^ T-helper cells [20, 34]. By ex vivo experiments, performed in an autologous human system, the ability of anti-wt p53_110–124_ CD4^+^ T cells to enhance the generation and antitumor function of CD8 effector cells was demonstrated. The results emphasize the crucial role of T helper-defined epitopes in shaping the immune response to multiepitope cancer vaccines targeting p53. This suggests that future vaccination strategies targeting tumor cells should incorporate helper and cytotoxic T cell-defined epitopes [34].

## 4. Mechanisms of Tumor Evasion

### 4.1. Suppression of T Cells in the Cancer-Bearing Host

Homeostasis of lymphocytes, that circulates through the tissues and the blood, is maintained by refreshing the pool via the thymic output of naive lymphocytes and expansion of antigen-specific lymphocytes upon adequate stimulation and contraction of the lymphocyte pool by death of lymphocytes in the periphery that have completed their functions [35–37]. 

One of several mechanisms by which tumors escape from the host immune system is induction of apoptosis in effector T cells [12]. It was shown that a proportion of CD3^+^, Fas^+^ T cells in the peripheral circulation were in the process of apoptosis. This indicates that the Fas/FasL pathway is involved in spontaneous apoptosis of circulating CD95 (Fas^+^) T lymphocytes. Interestingly, these cells showed decreased expression of CD3-zeta-chain. Expression of T cell receptor- or Fc-gamma receptor III-associated signal-transducing zeta chain is important for the functional integrity of immune cells [31]. Fas/FasL interactions might lead to increased turnover of T cells in the circulation and, consequently, to reduced immune competence in patients with SCCHN [38]. This may be explained by an imbalance in the absolute counts of T-lymphocyte subsets and an overall decreased absolute T cell count in patients not treated with cytotoxic agents [39, 40]. The rapid turnover affects mostly T cells with effector phenotype [41] that also show defects in signaling [31]. Preferentially tumor-specific T cells are affected by apoptosis indicating a tumor-related effect [10]. This observation can be explained further by the analysis of TCR Vbeta profiles of CD8^+^ T cells in patients with SCCHN that were altered relative to normal controls. This may reflect increased apoptosis of expanded or tumor-contracted CD8^+^ T cells, which define the TCR Vbeta profile of antigen-responsive T-cell populations in patients with cancer [19]. Reports on T-cell apoptosis at the tumor site and in the peripheral circulation [42, 43] support these observations and suggest that death of tumor-infiltrating lymphocytes (TIL), generally considered to represent tumor-associated antigen-specific effector cells, is driven by the tumor or tumor-derived factors. Recent studies in SCCHN also demonstrate that T regulatory cells (Treg) express high levels of Fas and selectively kill CD8^+^ T effector cells via Fas/FasL [44]. FasL is upregulated exclusively on Treg isolated from patients with no evidence of disease after receiving cancer therapy [44]. These FasL-expressing Treg are resistant to apoptosis themselves but strongly suppress and kill CD8^+^ effector cells, adverting the cancer community that traditional cancer therapy might contribute to tumor progression by collaborating with the peripheral tolerance process. In addition, Treg in patients with SCCHN kill CD4^+^ T effector cells via granzyme B in the presence of IL-2 [44]. Signaling defects in the TCR as well as NF-B activation pathways in TIL have been described in comparison to T cells infiltrating inflammatory noncancerous sites. These defects appear to be responsible for their loss of function [45]. Patients with tumors infiltrated by TIL expressing normal levels of CD3-zeta-chain were found to have a better 5-year survival than those showing loss of CD3-zeta-chain expression [8, 46]. A high rate of apoptosis in TIL is considered to be a factor for poor prognosis [47].

Taken together it appears that apoptosis of lymphocytes in the periphery as well as at the tumor site leads to rapid and selective tumor-specific lymphocyte turnover followed by a loss of effector cells and thus failure to control tumor growth in cancer patients.

### 4.2. Role of Regulatory T Cells

Because of the identification of the forkhead box transcription factor Foxp3 as an essential transcription factor in CD4^+^ regulatory T cells (Treg), Treg have been well characterized as a distinct lineage of T cells [48]. Their thymic origin (denominated naturally occurring Treg or nTreg) as well as their importance for the maintenance of peripheral tolerance under noninflammatory conditions throughout the life span of an individual, has been demonstrated in men and mice [49, 50]. When Foxp3^+^ Treg are depleted in an adult individual, fatal multiorgan autoimmunity finally results [51] and the phenotype of this disease is virtually indistinguishable from genetic deficiency of Foxp3 that is characterized by a massive lymphoproliferative syndrome [52, 53]. However, the conditions and mechanisms required to generate Foxp3^+^ Treg *de novo* in the peripheral immune compartment (denominated inducible Treg or iTreg) or to selectively expand Treg in peripheral blood and lymphoid organs are less clear. Essential roles of IL-2, TGF-*β*, and TCR signaling in iTreg generation, expansion, function, and survival have been established [54–56]. However, it is unclear which particular arm in each of these pathways is important in FoxP3 regulation. Foxp3 expression is required for suppressor capacity of Treg in men and mice [57]. In addition, regulation of Foxp3 expression in nTreg as well as iTreg in response to acute and chronic inflammation remains an unresolved issue. Elucidating the signaling pathways that command Foxp3 induction and expression in inflammation constitutes a challenge of particular interest as Treg induction, expansion, survival, and function are significantly altered in diseases in which inflammation is a key regulator of the pathology (i.e., autoimmune disease and cancer).

It has become increasingly clear that malignant transformation and cancer progression are immunologically recognizable events and the immunologic status of the host as well as the inflammatory conditions of the tumor microenvironment influences significantly the outcome of the patient [58]. In early stages, inflammation at the tumor site induces chemotaxis of immune cells from the periphery to the tumor and immunologic recognition may exert selective pressure, braking tumor growth of the emerging cancer [59]. However, when the immune system is confronted with persistent exposure to tumor antigens the establishment of tolerance [60] and in consequence immunosuppression of the patient are favored. Much like tolerance to normal self-antigens, tolerance to tumor antigens (TA) can arise from a failure to encounter antigen or the deletion or functional inactivation of tumor-specific T cells. It has been demonstrated during the last 10 years that Treg frequency increases in peripheral blood, lymph nodes, and tumors of patients with several types of cancer [61], including patients with HNSCC [62, 63]. It also correlates with tumor progression and outcome [64]. Suppressor capacity and suppressor phenotype of Treg isolated from SCCHN cancer patients are significantly increased in comparison to Treg isolated from healthy subjects [62, 63], suggesting that enhanced function and survival of suppressor cells might constitute one of the mechanisms that are responsible for immunosuppression of adaptive and innate immunity in these patients. Indeed, in several models, tumor immunosurveillance is augmented when CD4^+^CD25^+^ Tregs are depleted [65, 66]. Removing Treg has also been shown to increase tumor immunity elicited by vaccination [67].

Thus, one therapeutic possibility for restoration of anti-tumor immunity in patients with cancer is to eliminate tumor antigen (TA)-specific Treg and to boost simultaneously TA-specific T helper and CTL responses. The fact that Treg and activated T effector cells share receptors and common metabolites in their differentiation, function, survival, and expansion (i.e., IL-2) suggests that regulation of the effector and suppressor compartment is dichotomic. Thus, one new challenge in modern immunotherapy is to understand the signaling pathways that command the interplay of effector and suppressor responses in physiologic conditions and in inflammation. A detailed knowledge of these pathways might enable us to design immunotherapeutic strategies that selectively promote expansion, survival, and function of effector or Treg responses in pathologies where one of the two compartments is in disadvantage resulting in autoreactive killer responses in the absence of Treg or in immunosuppression in the case of an excessive Treg response. Revert immunosuppression in cancer to anti-tumor immunity is essential to increase the quality and success of traditional cancer therapy as well as the response to tumor vaccines.

Clinical trials for tumor vaccines using TA antigens and antigen presenting cells (APCs) or DC are now under way for the treatment of different types of cancer. To date, more than 1000 tumor vaccines have been reported [68]. The collective results are encouraging but not satisfying. The study of Chakraborty et al. shows that the MAGE-specific CTL response in patients with melanoma contracts in circulation by day 28 after vaccination [69]. Multiple factors may explain the decline of TA-specific CTL in circulation of patients after receiving a tumor vaccine. For example, the decline might be a result of the activated CTL leaving the circulation and homing into extravascular sites. The contraction might also be a reflection of programmed cell death or activation-induced cell death as physiologic homeostatic process [69]. However, the work of Zhou et al. suggests that contraction might also result from a Treg cell regulated process. They demonstrate that vaccination of the tumor-bearing host expands Treg, blunting the expansion of naïve tumor-specific T cells and blocking the execution of effector function *in vitro* and *in vivo* [70]. The reports of Zhou et al. and Chakraborty et al. suggest that the development of treatment paradigms that seek to not only increase the frequency of tumor-specific T cells but to do so in conjunction with strategies that selectively inactivate or remove suppressor T cells is a must. This objective has become even more complex with the recent identification of Th17 cells. Collectively, the amendment of the Th1/Th2 paradigm by new subsets of T cells has resolved some inconsistencies of the Th1/Th2 paradigm but has also made the understanding of the pathogenic process of cancer inflammation more complex. The fact that Th17 cell induction and differentiation is mediated by metabolites that are also essential in Treg development and function suggests that Treg-Th17 cell interactions might influence the induction process of peripheral tolerance. Indeed, some recent studies report that Th17 cells increase simultaneously with Treg in cancer subjects and this dichotomic increase correlates with cancer progression [71]. Thus, a new challenge in cancer immunology has become to elucidate the population dynamics and kinetics of Th17 and Th1 cells. Their interplay and susceptibility towards regulatory mechanisms at the site of inflammation and in the periphery possibly defines phase-specific approaches for therapeutic interventions in order to prevent cancer–inflammation-mediated tolerance.

First studies investigating the dynamics of Treg and effector responses indicate that molecular signaling pathways in response to IL-2, TGF-*β*1, and the TCR are determinant in regulating homeostasis of suppressor cell and effector cell populations [54–56, 79]. Therefore, a detailed knowledge of these pathways might provide valuable insights on how Treg might be regulated to support tumorrejection. 

IL-2 plays a dominant role *in vivo*, in the maintenance of immune system homeostasis and self-tolerance. The latter function is emphasized by the finding that mice deficient in IL-2 or components of the IL-2 receptor (IL-2R*α* or IL-2R*β*) succumb to lymphoproliferative autoimmune syndrome, with the effect of IL-2R*β* lack being more severe [80, 81]. TGF-*β*1 is a pluripotent cytokine that has pronounced effects on T cell-mediated immune suppression as well as on the control of autoimmunity. The role of TGF-*β* is in influencing the constitutive expression of Foxp3. IL-2, the transcription factor NF*κ*B, might be essential in regulating Treg and T effector cell homeostasis in conditions of inflammation. Elucidating the role of TGF*β* and NF-*κ*B in Treg and T effector cell dynamics is promising because both are up-regulated during cancer inflammation and are common regulators of Treg and Th17 cell differentiation. The design of studies that elucidate these signaling pathways in patients with cancer constitutes one step towards immunotherapeutic strategies that enforce immunogenicity of tumor vaccines.

Taken together, these complex immunoregulatory mechanisms lead to an immunoediting of the T cell and in particular the CTL response by the tumor in order to avoid elimination by the immune system. However, also the tumor cell immunogenicity is edited by the immunoresponse.

### 4.3. Tumor-Immune Escape

Tumor-T cell interaction leads to a negative selection pressure on tumor cells that are being recognized by the immune system. T cell recognition is therefore reduced in tumor cells by downregulation of molecules important for antigen processing and presentation or costimulation [82]. 

Several observations demonstrate an immune selection of SCCHN tumor cells as discussed below. This selection process and the resulting immune escape variants in the tumor indicate that an effective CTL response must have taken place during the development of the malignancy. The CTL-mediated cytolysis of immunogenic tumor cells is the driving force of the selection process towards non-CTL-susceptible tumor cell variants. The immune-evaded tumor cells have several features making them resistant to further natural CTL attack.

- Reduced expression of costimulating molecules on the cell surface leads to inadequate T-cell activation, even if the tumor is recognized and in turn to tolerance induction [83].
- Expression of MHC class I is altered in up to 50% of SCCHN [84–86]. Both, total loss of HLA-class I and more selective downregulation of the HLA-A, B, or C locus expression has been shown [87].
- Expression of components of the antigen processing machinery (APM), namely, LMP2, LMP7 and TAP1 is frequently downregulated or completely lost in tumor lesions as compared to surrounding tissue as well as in cell lines obtained from SCCHN cancer patients. As a consequence of the downregulated APM components, fewer antigen is processed and loaded onto MHC-complexes that are by themselves decreased in number or dysfunctional.

In combination, these three mechanisms compromise recognition of the progressed tumor by tumor-specific T cells. In cell lines, the expression of APM-components could be restored by incubation of with IFN-g [18, 88]. Furthermore, LMP2, LMP7, TAP1, TAP2, and HLA class I antigen expression rates in primary SCCHN lesions were found to predict overall survival [88]. Since expression of APM-components could be functionally restored, structural abnormalities such as genetic alterations are unlikely. With regard to the two different etiologies of SCCHN (one being alcohol and tobacco abuse and the other oncogenic human papillomavirus), more detailed studies are needed to investigate if this dysregulation can be observed in tumors with both etiologies. Whether this is a general phenomenon, as has been reported in other tumor types without viral etiologies, or is due to HPV specific factors, as has been suggested in HPV-6- and HPV-11-associated laryngeal papilloma [89], remains to be clarified. So far, only in one of our studies HPV-typing was carried out [18].

Tumors can also interfere with the immune system by producing and releasing numerous factors that modulate functions of immune cells or directly induce apoptosis. These factors take action in the tumor microenvironment and beyond.

## 5. Vaccination Strategies Aiming at Induction of Cytotoxic CTL and Reversing Immune-Escape Mechanisms

For effective vaccination, a successful stimulation of the immune system and an effective modulation of suppressive effects exerted by the tumor cells are necessary (Table 1).

### 5.1. Role of APM and Abnormal MHC Class 1 Expression

**Figure 1:**
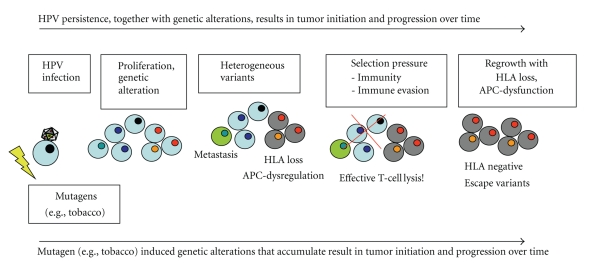
Immune escape variants of cells in the tumor indicate an effective T cell response. HPV infection leads to unregulated cell proliferation and accumulation of chromosomal aberration. Cumulative genetic alterations in tumor cell subclones lead to the emergence of tumor cell variants with divergent characteristics, for example, loss of HLA expression. Selection pressure is exerted by the microenvironment of the tumor and immune response mechanisms. Over time, susceptible cells will be eliminated and resistant cells will regrow, to form a tumor consisting of predominantly immunoresistant cells and compromising immunotherapeutic strategies.

Downregulation of dysfunction of APM components by the tumor may disturb both the induction of tumor-specific T cells in the initial phase of the immune response and subsequently during the effector-phase the proper recognition of the tumor. This effect is augmented by absent or reduced presence of MHC class-I molecules on the cellular surface. These cells are considered to have a more aggressive phenotype [90] which may also be the result of immunoselection of tumor cells able to evade the immunosurveillance (Figure 1). The result can be seen by a low number of tumor infiltrating lymphocytes and ineffective generation, activation, or even enhanced apoptosis of tumor-specific T cells [9, 10, 18, 91]. 

In experimental systems, incubation of SCCHN cell lines with IFN-*γ* was able to restore T cell recognition and killing [18, 88]. These preliminary data should inspire more basic and clinical research to better understand and further refine and develop these adjuvant strategies for clinical application. From the current point of view, it seems indispensable to combine APM- and MHC-class-I restoration with induction of tumor-specific immune responses.

### 5.2. Apoptosis of T Cells

Tumor-induced increased apoptosis of lymphocytes, including NK-cells and tumor-specific CD8^+^ T cells in the peripheral circulation and at the tumor site, has been observed [10, 38, 41–43, 92]. This phenomenon may lead to depletion of the lymphocyte pool from which tumor-specific effector cells could be recruited or expanded by immunotherapy. Therapeutic approaches should, therefore, aim to reverse this effect by restoring a normal lymphocyte turnover or protection of CD8^+^ T cells from apoptosis [93].

### 5.3. Monitoring and Targeting Treg Responses in Patients with Cancer: Therapeutic Relevance

The observation that Treg are increased in peripheral blood and at the tumor site in patients with cancer suggests that the development of treatment paradigms that seek to not only increase the frequency of tumor-specific T cells but to do so in conjunction with strategies that selectively inactivate or remove suppressor T cells is a must. However, the monitoring of Treg frequency and function in patients with cancer is not only required to improve the success of cancer vaccines and traditional cancer therapy but might also provide a broader basis for the development of more reliable prognostic factors, improving a tumor-free outcome after therapy. On the other hand, selective depletion of Treg in patients that have received traditional cancer therapy might be essential to avoid or decrease recurrent disease. We have shown that hematologic recovery in response to oncologic therapy results in expansion of the CD4^+^CD25^+^ compartment, including CD4^+^CD25^high^Foxp3^+^ [62]. Importantly, not only was the proportion of CD4^+^CD25^+^ T and CD4^+^CD25^high^Foxp3^+^ cells increased in the patients after receiving oncologic therapy, but suppressor function and survival of these cells were significantly elevated. The observed expansion of Treg expressing Foxp3 correlated with increased suppression mediated by these cells in patients with non evident disease [62]. The effect of oncologic therapy on Treg might be related to two phenomena: (i) homeostatic regulation after radio/chemotherapy-induced lymphopenia resulting in the expansion of a total lymphocyte pool, and (ii) activation and expansion of *de novo* induced Treg and T responder cells by inflammatory cytokines derived from the strong inflammatory response that usually accompanies radio/chemotherapy. The immune system controls the level and the activation state of each cellular compartment through homeostatic regulation, a process that is triggered during development and after the induction of a lymphopenic state by external stimuli [94]. Radio/chemotherapy may have profound effects on the peripheral blood cell count, due to an increase in the availability of homeostatic cytokines and increased interactions of T cells with APC. It has been proposed that lymphodepletion removes endogenous cellular elements that act as sinks for cytokines, which are responsible for augmenting the activity of tumor-reactive T cells [95]. Thus, T-cells surviving after therapy receive very strong stimuli (cytokines and enhanced APC-T cell interactions) that trigger T-cell activation and expansion. Several groups have reported that antitumor responses seen after adoptive transfer of tumor-reactive T cells into lymphodepleted hosts are significantly increased [96, 97]. In this context, it is reasonable to suggest that a similar mechanism (s) is involved in activation and expansion of the activated T responder and memory T-cell compartments in SCCHN patients who receive oncologic therapy. Concomitantly, the Treg subset is significantly expanded. These findings suggest that monitoring of Treg frequency and function as well as depletion of Treg after oncologic therapy might be crucial to allow the development of an effective antitumor T-cell response able to eliminate secondary tumors.

### 5.4. Single- and Multi-T-Cell Epitope Vaccines

p53 may serve as a model antigen for the development of broadly applicable antitumor vaccines in SCCHN. A number of p53-derived epitopes that can be used for the design of vaccines have been identified [98, 99]. Since mutations in the p53 sequence are frequent [100], epitopes incorporating these mutations would have to be tailored specifically to each patient. Therefore, epitopes composed of the wild-type (wt) sequence are especially attractive, since they are shared among the same HLA type and are therefore not patient specific. 

*In vitro* stimulation of CD8^+^ T cells with wt p53 peptide-pulsed autologous dendritic cells can be used to induce either HLA-A2-restricted, wt p53_149-157_ and/or wt p53_264-272_ peptide-specific responses from epitope-specific precursors. Interestingly, using these single epitopes, wt p53 peptide-specific CD8^+^ T cells were generated in only a third of healthy donors or subjects with cancer [27]. Others have reported comparable findings [101, 102]. The limited responsiveness of healthy donors may be explained by negative thymic selection of T cells with receptors specific for self-antigens. It can be expected that especially T cells with high-affinity receptors are eradicated. The observed limited responsiveness to HLA class I-restricted wt p53 peptides among HLA class I-compatible healthy donors and subjects with cancer suggests that multiple wt p53 peptides are needed in order to maximize donor responsiveness. The underlying causes can only be suspected and may partly be due to the mechanisms of tumor immune evasion discussed above. 

Since it may prove difficult to determine in an individual case the responsiveness prevaccination, a vaccine consisting of more than one epitope may be the more promising approach. 

### 5.5. Immune Intervention Approaches For HPV-Associated SCCHN

Oncogenic HPV genotypes, particularly the HPV types 16 and 18, are found in a subset of SCCHN mostly lacking the risk factors of alcohol and tobacco abuse. It therefore seems to be a suitable malignancy for vaccination against HPV-associated targets. Thus, these HPV-associated SCCHN may be preventing by (a) the existing prophylactic HPV-vaccines or (b) treated by vaccines designed to induce an appropriate antitumor immune response against HPV-specific tumor antigens [18, 22]. Lately, several authors have advocated a combined approach of prophylactic and therapeutic HPV-vaccination in patients with dysplasia and risk for reinfection with oncogenic HPV-types [103, 104].

**(a) Prophylactic HPV Vaccines**

Neutralizing antibodies specific for the viral capsid proteins may prevent infection by HPV. Prophylactic HPV vaccines have been developed and approved. As antigen, they contain capsid protein L1 of the most prevalent HPV types. They were introduced in 2006 and have the estimated potential to reduce the burden of cervical cancer, the tumor entity they were originally developed for, by approximately 70% [105, 106] and the vaccine type-related precancers. Estimations for SCCHN are currently not available. Prophylactic HPV vaccines have, however, no therapeutic potential due to their mechanism of action via virus-neutralizing antibodies targeting the L1 capsid antigen. Unfortunately, this capsid protein is not expressed in persistently HPV-infected basal epithelial cells and transformed cells in infected mucosa and is therefore useless for therapeutic vaccination. Accordingly, prophylactic vaccines have not shown any therapeutic activity [107].

However, they may be of benefit in posttherapeutic situations where infected lesions have been removed surgically to prevent formation of new lesions due to reinfection [104]. These settings are currently under investigation.

**(b) Therapeutic HPV Vaccines**

The rationale for vaccines targeting HPV-associated SCCHN is that virus-related oncogenes are obligatory to tumor growth. Vaccines with therapeutic potential must target the two HPV oncogenic proteins, E6 and E7 as antigens that are important in the induction and maintenance of cellular transformation and are coexpressed in the majority of HPV-associated carcinomas. Two studies have investigated if an endogenous T-cell immunity to HPV-encoded oncogenes E6 and E7 in SCCHN patients exists [18, 22]. This group of T cells would have the potential to specifically identify and target the tumor upon appropriate activation. Therefore, these cells are a critical prerequisite for the development of vaccine-based strategies for enhancing antitumor immunity in patients with HPV+ tumors. Indeed, in both studies it was found that infection with HPV-16 (as compared to uninfected control individuals) significantly alters the frequency and functional capacity of virus-specific T cells in SCCHN patients. In addition to the presence of HPV-specific effector T cells, successful tumor elimination requires that HPV-infected tumor cells function as appropriate targets for cytotoxic T lymphocyte recognition and elimination. Immunohistochemistry of HPV-16+ SCCHN tumors showed that the antigen-processing machinery components are downregulated in tumors compared to adjacent normal squamous epithelium [18]. Thus, immunity to HPV-16 E7 is associated with the presence of HPV-16 infection and presentation of E7-derived peptides on SCCHN cells, which shows evidence of immune escape comparable to cervical cancers [108]. These findings suggest that development of E7-specific immunotherapy in HPV-associated SCCHN should be combined with strategies to enhance the antigen processing machinery component expression and function [18].

## 6. T-Cell Therapies Directed to Cancer Stem Cells

**Figure 2:**
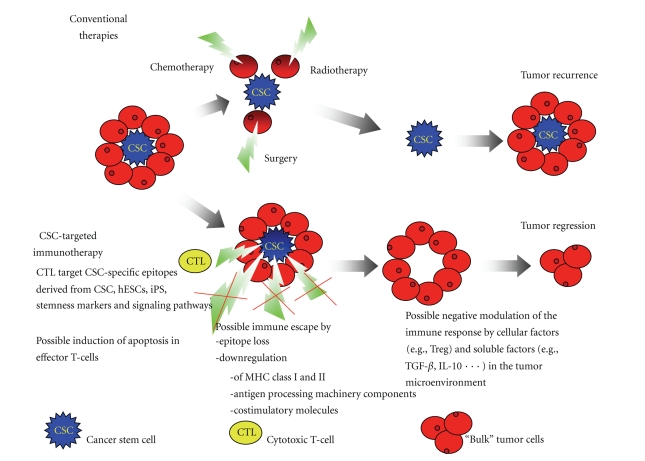
Comparison of the effects of a failed conventional therapy and the outcome of a hypothetical CSC-targeted immunotherapy. Currently applied conventional therapies target bulk tumor cells that are less resistant than CSC. This leads to initial shrinking of the tumor mass but eventually regrowth from residual CSC. An immunotherapeutic approach targeting CSC directly would cut off the rejuvenating supply of CSC and ultimately lead to tumor regression.

Tumors consist of heterogeneous populations of cells. According to the cancer stem cells (CSCs) hypothesis, CSCs are a subpopulation of the tumor more capable than other cells to self-propagate, initiate new tumors differentiate into bulk tumor, and therefore sustain tumor growth. Because of these properties, CSCs have been moved into the focus of targeted therapies. The current knowledge of the existence of CSCs begins to lead to studies of their specific elimination (Figure 2 and Table 2). It is being envisioned that the targeting of CSC in combination with the established therapeutic modalities such as radiation and chemotherapy that due to a relative resistance of the CSC more preferentially kill the bulk of the tumor may decrease the frequency of recurrences and enhance the patient's long-term survival. Therefore, the development of strategies that target the CSC population directly is highly desirable. Eliminating CSC leads to an abrogation of the replenishing pool of cancer cells and ultimately leads to petering out the tumor growth, as has been documented in animal experiments where removal of CSC and transplantation of only the non-CSC tumor cells did not lead to sustained tumor growth.

The development of CSC targeted therapy has to overcome three major hindrances, that relatively to the bulk tumor population are increased (i) chemoresistance, (ii) resistance to radiotherapy, and (iii) immunescape mechanisms.

Since radio- and chemotherapies have already been optimized towards the limits of clinical benefit and yet tolerable side effects, a very attractive alternative approach of specifically targeting CSC is to develop antitumor T cell vaccines. One of the possible reasons that these therapies lacked efficacy in past clinical trials could be attributed to the fact that rather bulk tumor than CSC have been targeted. This may change with the identification of tumor-specific epitopes derived from CSC markers. One such as CSC model-target for head and neck cancer and others is the recently described CD8 defined T-cell epitope of aldehyde dehydrogenase-1 (ALDH1) [73]. Examples of other such CD8 defined T-cell epitopes are available for prostate stem cell antigen [76]. Less well-defined approaches include the development of a CSC-dendritic cell vaccine [72]. Recent studies using animal models for prostate cancer and malignant glioma demonstrated the potential of different vaccination strategies (dendritic cells, gene-gun and virus) targeting CSC in cancer immunotherapy [74, 75]. It was suggested recently that stemness related proteins expressed in CSC might also be a source for tumor antigens. Tumor types most dependent on CSC for their growth kinetics were named to be the best suited for approaches targeting stem cell genes [109]. 

In several studies, the efficacy of potential therapies directed against stem cell-associated signaling pathways are tested. For example, T cell immunity against embryonic stem cells antigen SOX2 and SOX6 has been explored in glioma stem cells [77, 78]. Since the expression of stemness-related genes is a common feature of stem cells and CSC, the question of vaccine induced autoimmunity to stem cells will have to be addressed by scientists following this path. One example is the vaccination with embryonic stem cells (ES) or induced pluripotent stem cells (iPS) that has been shown to induce protective immunity in colon carcinoma [110]. Another group used dendritic cells (DC) generated from mouse and human ES or iPS as a means for anti-cancer immunotherapy [111]. 

Success of these potential therapies will depend on how well immunological responses to CSC can be modulated for example by vaccine adjuvants upregulating antigen processing and presentation. Recently, a reduced activity of the 26S proteasome in breast cancer cells and in gliomas was observed as a feature of CSC [112]. Proteasomes are thought to play an important role in antigen processing and presentation of antigens in association with HLA class I [113]. This may result in reduced antigen processing and presentation of peptides presented to the immune system on major histocompatibility complex -I molecules. Reduced proteasomal activity was also used as explanation for the high expression levels of known stem cell markers like BMI-1 and nestin in CSC, which are both substrates of the proteasome [114, 115].

## 7. Significance of CSC For Future Treatment Strategies

The classification of conclusive CSC markers followed by the identification of defined T cell-recognized CSC epitopes in the future may lead to the clinical application of anti-CSC vaccination strategies. Several approaches are currently being evaluated (Table 2). Whether targeted therapies directed against stem cell-associated signaling pathways, which may be activated in stem cells and in CSC, will be of clinical use or be limited by undesirable side effects in vivo remains so far unresolved.

## 8. Conclusion

Immunotherapy is considered an attractive treatment modality because it specifically targets the cancer avoiding or minimizing side effects. Ideally, its therapeutic effects will also reach distant metastasis and will be sustainable lasting beyond the presence of the cancer due to immunologic memory.

Therapeutic strategies should consider that SCCHN has at least two distinct etiologies. One is chronic alcohol and tobacco abuse and the other is related to oncogenic human papillomavirus infection and transformation. Both etiologies will differ significantly in the antigenic make up of the tumor cells based on presence of self or viral antigens, respectively. In both cases, however, immunotherapeutic approaches should aim at induction of adequate antigen processing and presentation by the tumor cells to become visible for the immune system as target. Furthermore, tumor induced immune dysregulation should be redirected in favor of tumor rejection and finally an adequate stimulation of effector T cells capable of in vivo expansion and survival in the tumor-microenvironment is thought critical for improving clinical results.

## Figures and Tables

**Table 1:** Tumor escape and potential reversal strategies by adjuvant treatment options.

Tumor evasion mechanism	Desired effect	Potential reversal strategy
Loss of intracellular proteasomal antigen processing, transport (TAP deletion) and MHC-loading (beta2-microglobulin deletion)	Restoration of antigen-processing and MHC-loading, for sufficient tumor-antigen presentation	Interferon-*γ* treatment of the tumor
Silencing of MHC genes	Restoration of MHC-expression	Interferon-*γ* treatment, application of hypomethylating agents
Loss of T cell costimulation (e.g., CD80/86, and CD54, CD58)	Restoration of costimulatory molecules	Toll-like receptor stimulation, interferon treatment
Unfavourable microenvironment for CTL-response	proinflammatory microenvironment for CTL-response	Application of immune response modifiers, suitable vaccine adjuvants, and induction of CD4 T helper cells

Role of T cells		

Too few tumor-specific T cells	Induction of more CTL with lytic activity, broader T cell response including CD4 T-helper cells	Specific CTL stimulation and expansion. Vaccination with single or multiepitope vaccines including MHC class I and II peptides. Induction and expansion of CD4 T-helper cells.
Loss of immunodominant tumor antigen	Direction of the immune response to other antigens or epitopes	Identification of optimal MHC-class I and II epitopes. Reexpression of the tumor-antigen
Suppressive Treg effects	Inhibition of deleterious T-cell effects	Modulation/reduction of Treg by pretherapeutic treatment with antibodies or preferentially Treg targeting chemotherapeutic agents
Tumor-induced T cell apoptosis	Rescue of apoptotic T cells	T cell protection by: (i) reversal of redox potential (ii) treatment with anti-apoptotic drugs (iii) blocking of proapoptotic molecules (e.g., CD95)

**Table 2:** Examples for studies targeting CSC with CTL.

Target	Tissue	Ref
Dendritic cells loaded with CSC as antigen source	glioblastoma	[72]
CD8 defined ALDH1-specific T-cell epitope	HNSCC	[73]
Vaccination with murine prostate stem cell antigen encoding cDNA	Murine prostate cancer	[74]
Dendritic cells loaded with neurospheres from brain glioma cells	Murine glioma	[75]
Identification of 2 CD8 defined prostate stem cell antigen-specific T-cell epitopes	Prostate cancer	[76]
Vaccination with defined human embryonic stem cells (hESCs) or induced pluripotent stem (iPS) cells	Colon cancer	[5]
CD8 defined SOX2-specific T-cell epitopes	Glioma	[77, 78]

## Abbreviations

APC: Antigen presenting cell
DC: Dendritic cell
IFN-g: Interferon-gamma
SCCHN: Squamous cell carcinomas of the head and neck
HLA: Human leukocyte antigen
MHC: Multihistocompatibility complex.
